# Bat Colony and Cave Zone Shape Arthropod Assemblages in Levantine Caves

**DOI:** 10.3390/insects17010118

**Published:** 2026-01-21

**Authors:** Zeana Ganem, Shlomi Aharon, Dror Hawlena, Efrat Gavish-Regev

**Affiliations:** 1 The National Natural History Collections, Faculty of Sciences, The Hebrew University of Jerusalem, Edmond J. Safra Campus, Giv’at Ram, Jerusalem 9190401, Israel; shlomi.aharon1@mail.huji.ac.il; 2 The Department of Ecology, Evolution and Behaviour, The Institute of Life Sciences, Faculty of Sciences, The Hebrew University of Jerusalem, Edmond J. Safra Campus, Giv’at Ram, Jerusalem 9190401, Israel; dror.hawlena@mail.huji.ac.il; 3 Science Division, Israel Nature and Parks Authority, Jerusalem 95463, Israel; 4 The Department of Entomology, The Institute of Environmental Sciences, The Robert H. Smith Faculty of Agriculture Food and Environment, The Hebrew University of Jerusalem, Rehovot 7610001, Israel

**Keywords:** Arachnida, Arthropoda, assemblage composition, guano, habitats, subterranean

## Abstract

Caves are characterized by unique abiotic conditions such as limited light, and they therefore support distinct faunal assemblages that often include endemic species. Due to light limitations, photoautotrophic organisms are absent from many subterranean food-webs, which therefore predominantly rely on allochthonous nutrient sources. For this reason, hypogean habitats are expected to display lower assemblage diversity than that seen in epigean ecosystems. Bat guano, a major source of allochthonous nutrients in caves, varies substantially in composition based on its origin—whether it is produced by frugivorous or by insectivorous bats—and on its deposition site within the cave. This study examines how allochthonous nutrient sources and zones within caves influence arthropod diversity and assemblage composition. We found that both the type of allochthonous nutrient source and cave characteristics strongly affect the composition of arthropod assemblages. Our results show that caves harboring frugivorous bat colonies have a lower abundance of flies than caves with either insectivorous bat colonies or no bat colonies. Moreover, caves without bat colonies were seen to have low species richness of both detritivores and predators compared to caves housing either frugivorous or insectivorous bats. Additionally, species diversity and assemblage composition differed substantially between the twilight and dark zones of the caves. These findings demonstrate that allochthonous nutrient sources, the ecological zone, and the microhabitat within the cave are key drivers of arthropod assemblage composition and diversity. This study advances our understanding of cave ecology and underscores the importance of conserving diverse cave types for protecting their unique arthropod diversity.

## Simple Summary

Caves are unique environments, markedly distinguished from above-ground habitats by the absence of light in their deeper regions. Life in most caves relies on external nutrient subsidies, since photosynthetic organisms, which form the basis of most food-webs, cannot grow in the absence of light. Bat droppings, or guano, constitute an important nutrient source that supports unique food-webs in caves. In this study, we explore how different types of bat guano (from fruit-eating vs. insect-eating bats) and different zones within a cave (twilight vs. deeper regions) affect the number and type of arthropods, such as insects, spiders, and woodlice, inhabiting the cave. Our results indicate that caves with bat colonies contain more predators and decomposers than caves without bats. Additionally, caves hosting fruit-eating bats contained fewer flies than those inhabited by insect-eating bats and those lacking bat colonies. Moreover, the microhabitat within the cave is of importance: certain areas, such as the cave walls, support fewer species. This research highlights the role that bats play in controlling key ecosystem processes in caves. By demonstrating the important role played by bats in supporting underground arthropod life, this study emphasizes the necessity of protecting both bats and caves in order to preserve the hidden biodiversity of caves.

## 1. Introduction

Hypogean habitats are unique ecosystems defined by the reduced or complete absence of light, scarcity of nutrient sources, stable climatic conditions, and limited connectivity to surrounding habitats [1,2]. These conditions act as strong ecological filters restricting cave colonization and persistence of species in the underground [2,3]. The resulting hypogean assemblages display reduced species richness compared to epigean environments and are often composed of highly specialized and endemic taxa with unique adaptations to darkness [4,5,6]. The three ecological zones, also referred to as environmental zones, sometimes differ in various environmental conditions, including light intensity, temperature, air composition, and energy and nutrient sources, shaping species assemblages unique to each zone [3,5,6,7,8]. The entrance zone is characterized by high light intensity and greater connectivity to the external environment, and is mainly inhabited by visitor species that have no specific adaptations to subterranean life [7,9,10]. The twilight zone is characterized by reduced light intensity and serves as a transitional area inhabited by troglophile species, i.e., species that can survive both inside and outside caves, but other cave-adapted species may also occur in this zone. The dark zone is completely devoid of light and fosters stable climatic conditions that enable the presence of mainly troglobite species, which have unique adaptations to subterranean life [7,11,12]. These ecological zones include various microhabitats, each of which supports a different species assemblage [13]. For example, web-building spiders and flies typically occur on walls and cave ceilings, while cursorial predators such as ground spiders, pseudoscorpions, harvesters, and centipedes, as well as detritivores such as millipedes and isopods, are found mainly on the cave floor and under stones, particularly in detritus-rich areas. These microhabitats can facilitate the coexistence of multiple predator species within a single ecological zone [13].

The absence of light in hypogean ecosystems prevents photosynthesis, precluding the establishment of photoautotroph-based trophic chains [2,14]. In some exceptional caves, chemoautotrophic producers occur [15,16]. These chemoautotrophs do not rely on solar energy for synthesis, but rather provide additional energy to subterranean food-webs via processes such as sulfur compound oxidation [3,16]. These systems are characterized by simple food-webs [3] devoid of photoautotrophs and herbivores and by low species richness [14]. Most caves, however, rely predominantly on allochthonous organic matter and its decomposition by detritivores such as isopods, millipedes, and collembola, as well as by microorganism communities. The reliance on external organic inputs reduces the potential for energy transfer and nutrient cycling compared to epigean ecosystems, leading to simplified food-webs with fewer trophic levels. Epigean ecosystems include primary producers, herbivores, and other trophic levels, which generate assemblages of higher complexity and functional diversity [3,14]; for example, troglobite spider communities from caves in the Iberian Peninsula exhibit lower functional and alpha diversity than epigean spider communities [17]. The simplified food-web, reduced number of trophic levels, and absence of primary producers and herbivores lead to the production of detritus-based subterranean food-webs characterized by bottom-up control [18], as inferred from the composition of communities associated with allochthonous organic matter [19].

Bat guano deposited below bat colonies is considered a major source of allochthonous organic material in caves [4] and supports rich arthropod food-webs [20]. The nutritional composition of bat guano varies depending on whether bats are insectivorous or frugivorous [21]. In light of the high protein content of their prey, insectivorous bat guano is likely to be particularly rich in nitrogen [21,22]. In contrast, frugivorous bat guano is expected to have a higher carbon-to-nitrogen ratio (C:N), reflecting the high concentration of simple sugars in ripe fruits [21]. This variation in allochthonous nutrient sources has been hypothesized to lead to different arthropod assemblages in caves inhabited by either insectivorous or frugivorous bats [20,23]. Levantine caves are often inhabited by colonies of either insectivorous or frugivorous bats, although some are inhabited by both insectivores and frugivores, albeit in different zones of the cave. A recent study proposed that the spider assemblages of Levantine caves are shaped by several environmental variables such as the presence of bat guano in the cave and the climatic region in which the cave is situated [4,5]. A multivariate analysis of spider assemblages in Levantine caves suggested that high guano levels are associated with higher assemblage richness, particularly of troglobite and troglophile species, compared to caves with low guano levels or without guano [4].

While recent studies have been aimed at in-depth exploration of cave invertebrate fauna, and especially arachnofauna, around the world [4,5,24,25,26,27], the effect of allochthonous nutrient sources on arthropod assemblages remains largely unknown. This is particularly true with regard to the effect of different guano types (insectivorous bat guano vs. frugivorous bat guano) on arthropod assemblage composition (see [21,28,29,30,31]). Our study aims to bridge this knowledge gap by examining the way in which nutrient source type and cave zone interact to affect arthropod assemblages in Levantine caves. Specifically, we compare arthropod diversity and assemblage composition across different nutrient sources (caves with frugivorous bat colonies, caves with insectivorous bat colonies, and caves without bat colonies) and cave zones (twilight and dark). We also explored how these assemblages differ among microhabitats (on the cave wall, near the wall, and far from the wall) within the same ecological zone. We hypothesize that caves inhabited by bats will support arthropod assemblages of comparatively high diversity, owing to the nutrient subsidies provided by bat guano. Specifically, caves without bats are expected to have lower arthropod abundance and species richness than caves with bats due to the absence of guano as an energy and nutrient subsidy. Furthermore, caves with frugivorous bat colonies are expected to support higher arthropod species richness and abundance than those with insectivorous bats, owing to the energy-enriched basal source [32]. In addition, we hypothesize that bat roosting behavior, i.e., the zone in which the bat colony is found and the location of guano piles, will affect arthropod assemblage composition in the different cave zones. In the Levant, insectivorous bats typically roost deep in the dark zone of caves and deposit nitrogen-rich guano in localized patches. In caves inhabited by insectivorous bats, this spatial distribution of the guano may support higher arthropod abundance and predator richness in the dark zone relative to the twilight zone. By contrast, frugivorous bats often use the entrance and twilight zone for roosting and create much larger and more evenly distributed patches of bat guano. This variation may also affect the differences in arthropod species assemblages among zones in caves inhabited by insectivorous or frugivorous bats. We therefore hypothesize a resulting strong zone effect in caves dominated by insectivorous bats, with arthropod biomass, predator richness, and trophic complexity being substantially higher in the dark zone than in the twilight zone. In contrast, we expect a weaker or absent zone effect in caves dominated by frugivorous bats, with higher richness and higher activity of detritivores/omnivores across zones. In caves without bats, we expect less diverse assemblages with greater similarity among ecological zones.

## 2. Materials and Methods

### 2.1. Study Sites

**Figure 1:**
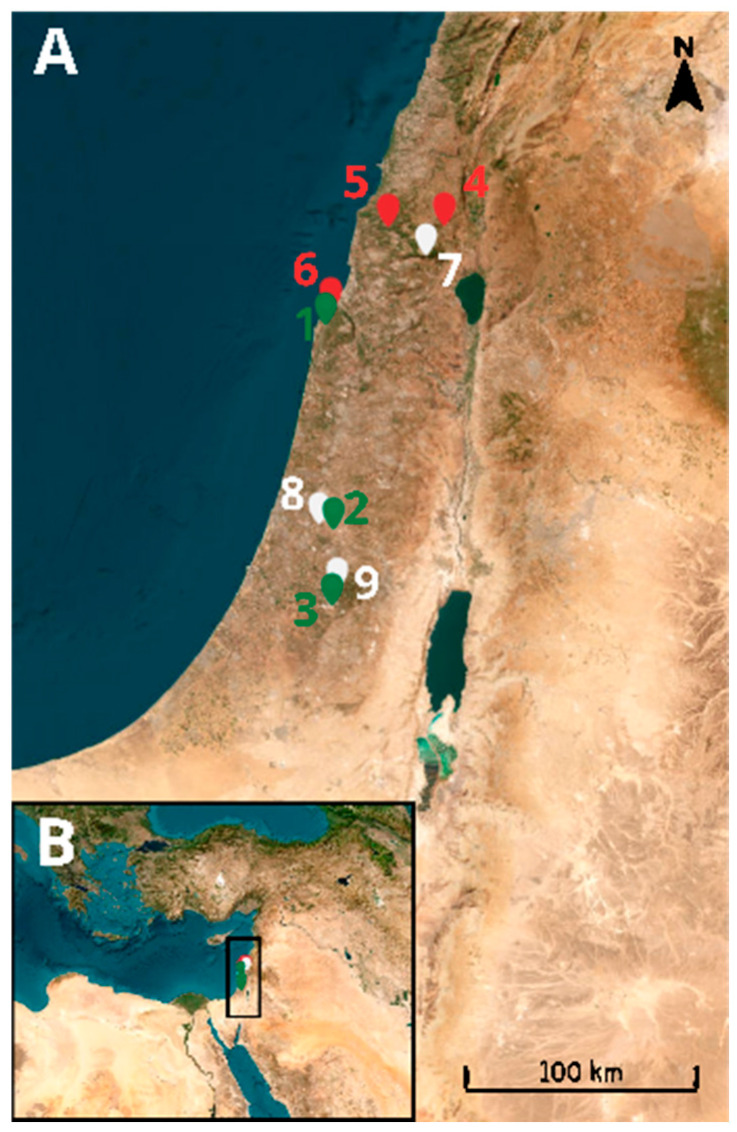
(**A**): Distribution of the studied caves. Red markers indicate caves inhabited by insectivorous bats. Green markers represent caves inhabited by frugivorous bats and white markers denote caves without bats. (**B**): Map of the Mediterranean Basin, with a rectangle highlighting the study area enlarged in panel (**A**).

**Figure 2:**
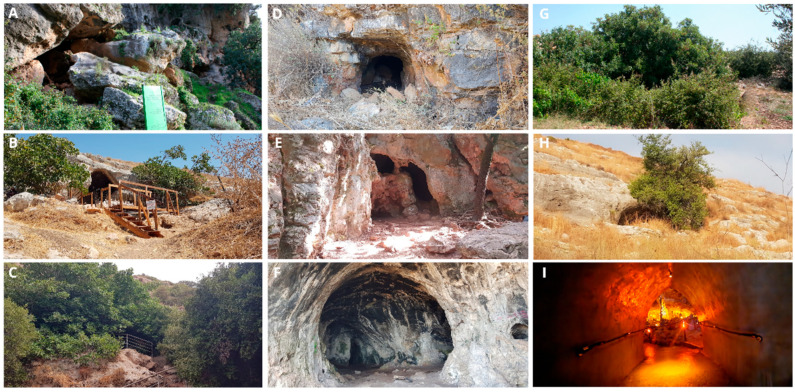
Openings of the caves in the study: (**A**–**C**): Caves inhabited by frugivorous bats. (**A**): Sefunim cave; (**B**): Tinshemet cave; (**C**): Te’omim cave; (**D**–**F**): caves inhabited by insectivorous bats (**D**): Yir’on cave, (**E**): Sharakh cave, (**F**): Ornit cave; (**G**–**I**): caves without bats; (**G**): Bet Jan cave; (**H**): Bet A’rif cave; (**I**): Soreq cave (the last is a show cave, with an artificial opening sealed by a door). Pictures: (**A**,**G**): Boaz Langford, (**B**–**F**,**H**,**I**): Zeana Ganem.

We conducted a field survey in August and September of 2021 in nine caves in the Mediterranean region of Israel. All of the caves contained both twilight and dark ecological zones [5]: three caves were inhabited by frugivorous bats (Sefunim (1), Tinshemet (2), and Te’omim (3)); three caves harbored insectivorous bats (Yir’on (4), Sharakh (5), and Ornit (6)); and three had no bat colonies (Bet Jan (7), Bet A’rif (8), and Soreq (9)) (Figure 1 and Figure 2). We recorded the temperature and humidity in both ecological zones of each cave using EXTECH Heat Index Psychrometer (Model RH25; Extech Instruments, Nashua, NH, USA) (see Appendix A, Table A1). Additionally, we noted the geographical coordinates, elevation, cave length, and cave opening size (see Appendix A, Table A1) for each cave. For estimating the cave opening size, we multiplied its height by its width. Cave length was estimated from maps provided by the Israel Cave Research Center.

### 2.2. Arthropod Sampling

In the twilight and dark ecological zones of each cave, we sampled three plot types representing different microhabitats: ‘center’ (sampling directly on the cave floor, far from the cave walls), ‘near the wall’ (sampling directly on the cave floor, where one of the plot edges is the cave wall), and ‘on the wall’ (sampling directly on the cave wall). We surveyed four 0.5 m × 0.5 m plots in each one of the three microhabitats in the two zones of each cave, for a total of 24 plots per cave. In each plot, we recorded all arthropods found during a five-minute visual search by two experts. All arthropods collected alive were kept in tubes placed in a cooler until transfer to the laboratory, where they were classified taxonomically and identified to the deepest possible taxonomic level. Individuals that were confidently identified in the cave were counted, listed, and released unharmed. All arachnids were identified using identification keys from the Araneae–Spiders of Europe website [33], Field Guide to the Spiders of Britain and Northern Europe [34], and a pseudoscorpion key [35]. Arachnids were also classified into one of three ecological categories (visitor/trogloxene, troglophile, or troglobite) based on published classifications and regional cave fauna literature [4,35]. Juvenile spiders were identified, when possible, by their family or genus, while adults were identified by their species or morphospecies. The remainder of the arthropods were identified to the deepest taxonomic level possible with the help of expert taxonomists. All collected arthropods were deposited at the National Natural History Collections, The Hebrew University of Jerusalem.

### 2.3. Statistical Analysis

We used four datasets to analyze and visualize diversity and assemblage composition. The first dataset included all arthropod samples identified to the order level. In the other three datasets, arthropods were identified to deeper taxonomic levels: arachnid families, arachnid species, and non-arachnid arthropod morphospecies. We generated rank abundance curves for these four datasets (except for arthropod morphospecies without arachnids) and Venn diagrams to visualize taxon overlap among cave categories. Because arthropod abundance data were highly skewed and contained extreme values, we report central tendency using median and 90% quantile ranges throughout the main text, unless otherwise specified. Means ± SD are presented only when directly required to compare with previous literature.

We tested whether (a) arthropod total abundance, (b) arthropod species richness (based on morphospecies), (c) troglophile arachnid species richness, and (d) troglobite arachnid species richness differ among cave types (inhabited by frugivorous bat colonies, insectivorous bat colonies, or without bat colonies), ecological zones (twilight or dark), and microhabitats (‘on the cave wall’, ‘near the cave wall’, and in the ‘center’) using Generalized Linear Mixed Models (GLMMs) in JMP Pro 17 (version 17; JMP Statistical Discovery LLC, Cary, NC, USA). on the entire dataset and on the richness of troglobite and troglophile arachnids using Poisson distribution and log link. Cave identity (Cave ID) was included as a random intercept effect to account for the non-independence of plots nested within the same cave.

We deployed multivariate analysis using direct ordination (CCA in Canoco [36,37,38]) to test the effect of 11 variables (df = 10) on arthropod assemblage composition. We used a combined dataset including our original “all arachnids” dataset and an additional 704 specimens randomly collected from the nine caves. For each cave, data from all plots within each ecological zone were pooled, resulting in a total of 18 samples, or 2 samples per cave. The eleven variables include eight continuous variables (elevation, latitude, longitude, length of the cave, size of the opening, temperature, humidity, and the total number of individuals in the sample) and one categorical variable with three levels (energy source: frugivorous bat guano, insectivorous bat guano, and other non-guano sources). In Canoco, each level of the categorical variable was tested separately. Each main effect was tested separately, while the remaining variables were set as co-variables (partial analysis). We used 99,999 unrestricted permutations.

## 3. Results

### 3.1. Arthropod Abundance and Richness by Cave Type, Zone, and Microhabitat

**Figure 3:**
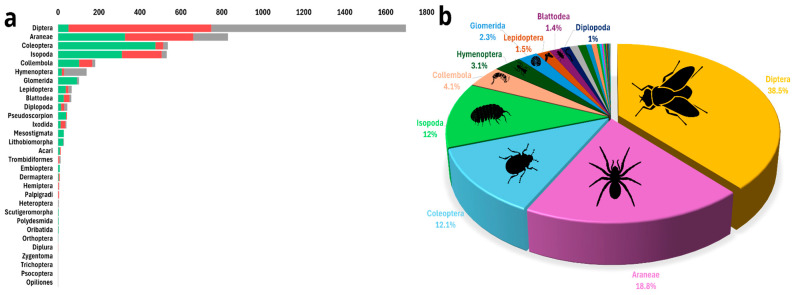
Arthropod assemblages. (**a**) Rank abundance graph of arthropod orders found across Levantine caves, by cave type: arthropods from caves inhabited by frugivorous bats in green, arthropods from caves inhabited by insectivorous bats in red, and arthropods from caves without bats in gray. (**b**) Relative abundance of all arthropod orders recorded across all caves, with only the ten most abundant orders labeled.

A total of 4411 arthropod specimens were recorded from the nine caves, of which 1280 were collected and transferred to the laboratory for further analyses; the remainder was identified in situ and released. We observed a total of 30 orders. We present the results here in order of abundance of taxonomical order. Diptera (flies) were the most abundant arthropod order observed (Frugivorous, 2.0 ± 4.0; Insectivorous, 16.6 ± 764.5; No bats, 19.8 ± 3739.6), with 1699 specimens belonging to 12 families. The extremely high standard deviation (SD) values reflect the extremely patchy distribution of fly aggregations in some caves. Because of this strong right-skew, we rely primarily on medians and quantile ranges for data interpretation (Table 1, Figure 3). However, flies were not distributed evenly among cave types; dipterans accounted for 69.4% of all arthropods in caves without bat colonies, 48.4% of all arthropods in caves inhabited by insectivorous bats, and only 3.2% of all arthropods in caves inhabited by frugivorous bats (Table 1, Figure 3). The five most abundant dipteran families were Limoniidae Latreille, 1802 (60 individuals); Dolichopodidae Latreille, 1809 (45 individuals); Phoridae Curtis, 1833 (16 individuals); Psychodidae Latreille, 1796 (13 individuals); and Mycetophilidae Newman, 1834 (10 individuals) (Table S1).

Araneae (Frugivorous, 2.0 ± 7.0; Insectivorous, 3.0 ± 20.7; No bats, 1.4 ± 1.2), the second most abundant arthropod order (830 individuals, representing 18.8% of all arthropods observed), will be discussed below, as this is the order for which the most taxonomic information is available.

The third most abundant arthropod order was Coleoptera (beetles) (Frugivorous, 9.2 ± 147.4; Insectivorous, 2.0 ± 1.9; No bats, 1.8 ± 5.2), with 536 specimens belonging to nine families. Like dipterans, beetles were not distributed evenly among cave types; they accounted for 29.7% of all arthropods in caves inhabited by frugivorous bats, 2.6% in caves inhabited by insectivorous bats, and only 1.6% of all arthropods in caves without bat colonies (Table 1, Figure 3). The four most abundant coleopteran families were Ptinidae Latreille, 1802 (167 individuals); Tenebrionidae Latreille, 1802 (79 individuals); Staphylinidae Latreille, 1802 (26 individuals); and Carabidae Latreille, 1802 (19 individuals).

The fourth most abundant arthropod order was Isopoda (Frugivorous, 10.1 ± 184.7; Insectivorous, 5.1 ± 86.8; No bats, 2.8 ± 24.7), with 531 specimens belonging to four families. As with other groups, isopods were not evenly distributed among cave types; they accounted for 19.5% of all arthropods in caves inhabited by frugivorous bats, 13.5% of caves inhabited by insectivorous bats, and in caves without bat colonies, they made up only 1.8% of all arthropods (Table 1, Figure 3). Most isopods that we found belonged to the family Porcellionidae Brandt & Ratzeburg, 1831 (355 individuals); the rest belonged to Armadillidae Brandt, 1831 (14 individuals), Trichoniscidae G.O. Sars, 1899 (13 individuals), and Trachelipodidae Strouhal, 1953 (14 individuals).

Other arthropod orders were less abundant, a total of 815 specimens, with Collembola (springtails) more abundant in caves inhabited by bats than in caves with no bat colonies (Frugivorous, 8.6 ± 209.4; Insectivorous, 4.0 ± 21.9; No bats, 1.2 ± 0.3), Hymenoptera more abundant in caves without bats (Frugivorous, 1.9 ± 4.8; Insectivorous, 1.4 ± 1.3; No bats, 7.9 ± 114.1), and Glomerida (pill millipedes) more abundant in caves inhabited by frugivorous bats than in other cave types (Frugivorous, 7.7 ± 39.2; no bats, 2.5 ± 5.7) (Table 1; Figure 3; Table S1).

**Figure 4:**
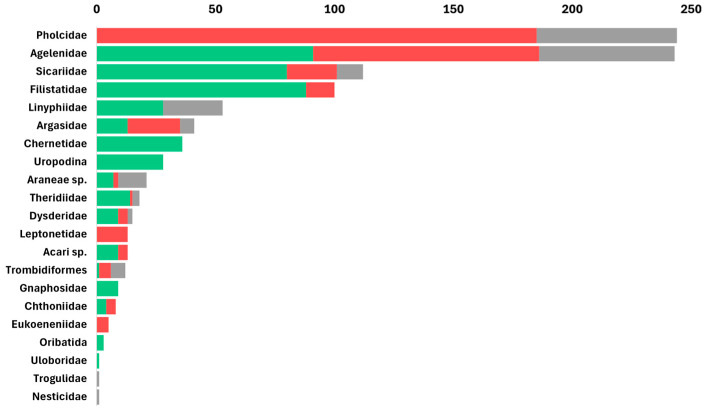
Rank-abundance graph of arachnid families and other higher taxa found across Levantine caves by cave type: arthropods from caves inhabited by frugivorous bats in green, arthropods from caves inhabited by insectivorous bats in red, and arthropods from caves without bats in gray. The taxa are sorted by their total abundance in caves. Spider (Araneae) families: Pholcidae, Agelenidae, Sicariidae, Filistatidae, Linyphiidae, Theridiidae, Dysderidae, Leptonetidae, Gnaphosidae, Uloboridae and Nesticidae; Ticks (Ixodida): Argasidae; Pseudoscorpiones families: Chernetidae, Chthoniidae; Acari: Mesostigmata: Uropodina; Trombidiformes; Oribatidae; acari unidentified; Palpigradi: Eukoeneniidae; Harvestman (Opiliones) families: Trogulidae.

**Figure 5:**
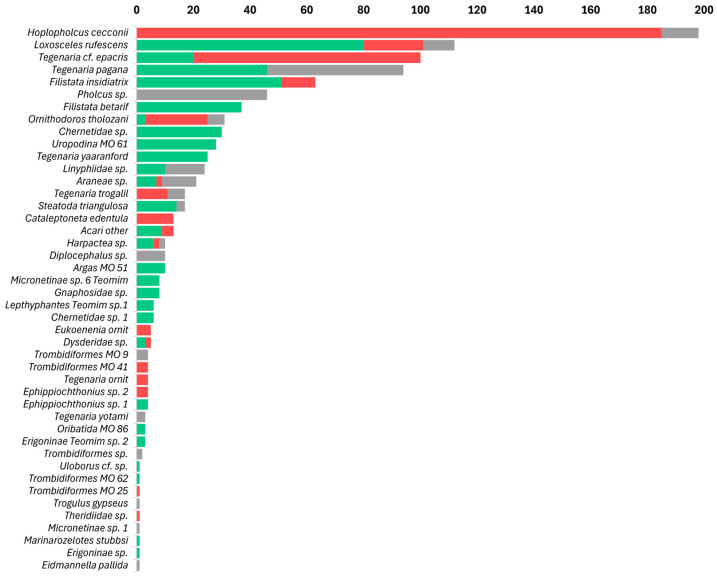
Rank-abundance graph of arachnid species found across Levantine caves by cave type: arthropods from caves inhabited by frugivorous bats in green, arthropods from caves inhabited by insectivorous bats in red, and arthropods from caves without bats in gray.

Arachnids, particularly the order Araneae, were the most abundant cave predators, with 977 individual arachnids recorded out of a total of 4411 arthropods (22.1%). In caves inhabited by frugivorous bats, arachnids constituted approximately 26.2% of all arthropods; in caves inhabited by insectivorous bats, arachnids constituted approximately 25.9% of all arthropods. In contrast, in caves without bats, arachnids represented 13.3% of all arthropods (Table 1, Figure 3). The 977 observed arachnids included representatives of 11 Araneae families and of 8 other arachnid orders, such as acari (Figure 4); these are presented here in decreasing order of abundance: the most abundant Araneae families were Pholcidae C. L. Koch, 1850 (244 individuals), Agelenidae C. L. Koch, 1837 (with 243 individuals), Sicariidae Keyserling, 1880 (112 individuals), Filistatidae Ausserer, 1867 (100 individuals), and Linyphiidae Blackwall, 1859 (53 individuals), which accounted for 76.9% of the total arachnid specimens. The rest (225 individuals) belonged to Argasidae (Ixodida; 41 individuals), Chernetidae (Pseudoscorpiones; 36 individuals), Uropodina (Acari; 28 individuals), Theridiidae (Araneae; 18 individuals), Dysderidae (Araneae; 15 individuals), Leptonetidae (Araneae; 13 individuals), Trombidiformes (Acari; 12 individuals), Gnaphosidae (Araneae; 9 individuals), Chthoniidae (Pseudoscorpiones; 8 individuals), Eukoeneniidae (Palpigradi; 5 individuals), Oribatida (Acari; 3 individuals), Uloboridae (Araneae; 1 individual), Trogulidae (Opiliones; 1 individual), and Nesticidae (Araneae; 1 individual). Twenty-one juvenile spiders that could not be identified, and thirteen mites (Acari) were not assigned to a family (Figure 4). In total, we identified 42 arachnid species and morphospecies (Figure 5). The five most abundant species were spiders belonging to four families: *Hoplopholcus cecconi* Kulczyński, 1908 (Araneae, Pholcidae, 198 individuals), *Loxosceles rufescens* Dufour, 1820 (Araneae, Sicariidae 112 individuals), *Tegenaria* cf. *epacris* Levy, 1996 (Araneae, Agelenidae 100 individuals), *Tegenaria pagana* C. L. Koch, 1840 (Araneae, Agelenidae 94 individuals), and *Filistata insidiatrix* Forsskål, 1775 (Araneae, Filistatidae 63 individuals) (Figure 5; Table S1).

a. Arthropod abundance

We found a significant interaction between cave type and ecological zone (Generalized Linear Mixed Model, F = 211.7, *p* < 0.0001), as well as a significant effect of the microhabitat (F = 79.5, *p* < 0.0001) on total abundance of arthropods in caves: the highest abundance was found in the twilight zone of caves with frugivorous and insectivorous bat colonies (median: 22, 21; 90% quantile: 63.7 and 77, respectively) and the lowest in caves without bat colonies (median: 6; 90% quantile: 43.5). In cave dark zones, the highest abundance was found in caves with frugivorous and insectivorous bat colonies and the lowest in caves without bat colonies (median: 8, 5, 3; 90% quantile: 59.5, 12.3, 29.6, respectively). Arthropod abundance was, on average, lower ‘on the wall’ than in the ‘near the wall’ and ‘in the center’ microhabitats of the cave (median: 5.5, 9, 10; 90% quantile: 67.3, 48.3, 52.3, respectively) (Table 2). Certain taxa, however, present a different picture: dipterans were more abundant in the ‘on the wall’ microhabitat (1175 individuals) than in the ‘center’ (172 individuals) and ‘near the wall’ (193 individuals) microhabitats.

b. Arthropod species richness

Cave type (F = 53.8, *p* < 0.0001), ecological zone within the cave (F = 18.4, *p* < 0.0001), and microhabitat (F = 30.6, *p* < 0.0001) were all found to have significant effects on arthropod species richness in caves; no interaction was found. A slightly but significantly higher species richness was found in caves housing frugivorous bat colonies than in caves with insectivorous bat colonies or without bat colonies (median: 4, 3, 3; 90% quantile: 9.7, 6, 5, respectively), and in twilight zones compared to dark zones (median: 4, 3; 90% quantile: 8, 6, respectively). Arthropod richness was lower ‘on the wall’ than ‘near the wall’ or in the ‘center of the caves’ (median: 2.5, 4, 4; 90% quantile: 5, 7.3, 7.3, respectively) (Table 2). A closer look at microhabitat specialists highlights these patterns: species such as *Alphitobius MO 12.* (Coleoptera, Tenebrionidae, 74 individuals), *Uropodina MO 61* (Acari, Mesostigmata 28 individuals), and Micronetinae Teomim sp. 6 (Araneae, Linyphiidae, 8 individuals) were dominant only in the microhabitat ‘center’. The ‘near the wall’ microhabitat was dominated by the Porcellionidae sp. (Isopoda, 141 individuals) and Formicidae sp. (Hymenoptera, 93 individuals); the species Limoniidae MO 102 (Diptera, 31 individuals) was found only in the ‘on the wall’ microhabitat.

c. Arachnid troglophile species richness

Cave type (F = 15.5, *p* < 0.0004) and ecological zone within the cave (F = 10.6, *p* < 0.0011) were found to have significant effects on arachnid troglophile species richness in caves; no interaction was found. A slightly but significantly higher arachnid troglophile species richness was found in caves with frugivorous bat colonies and caves with insectivorous bat colonies than in caves without bat colonies (median: 1, 1, 0.5; 90% quantile: 3, 2, 2, respectively). We found a similar median number of troglophile arachnid species in the twilight and dark zones (median: 1, 1); the maximum number and 90% quantile were higher, however, in the twilight zone (4, 3 and 3, 2, respectively) (Table 2).

d. Arachnid troglobite species richness

We found a significant interaction between cave type and ecological zone (F = 8.1, *p* < 0.0171) affecting arachnid troglobite species richness in caves. A slightly but significantly higher arachnid troglobite species richness was found in the dark zone than in the twilight zone of caves with insectivorous bat colonies (Maximum 4), followed by dark and twilight zones of caves with frugivorous bat colonies (Maximum 2) (Table 2).

Most of the troglobite arachnids we found are one-cave endemics, for example, Uropodina MO 61, Micronetinae Teomim sp. 6, *Lepthyphantes* Teomim sp.1, *Argas* MO 51, Micronetinae sp. 1, Chernetidae sp. 1, Oribatida MO 86, *Eukoenenia* sp. ornit, Erigoninae Teomim sp. 2, Trombidiformes MO 9, and Trombidiformes MO 41.

### 3.2. Arthropod Assemblage Composition and Species Overlap

**Figure 6:**
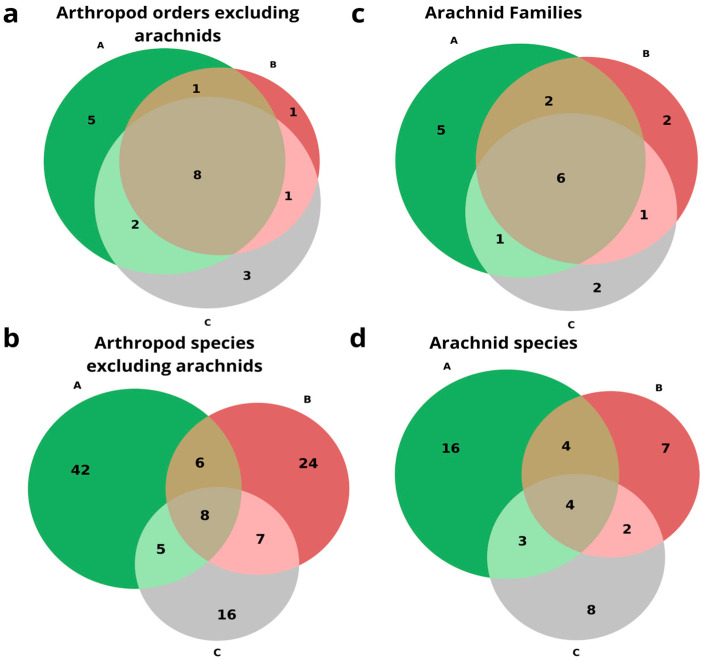
Venn diagram of the number of unique and shared (**a**) arthropod orders excluding arachnids, (**b**) arthropod species excluding arachnids, (**c**) arachnid families, and (**d**) arachnid species and morpho-species. A. Arthropods from caves inhabited by frugivorous bats in green; B. Arthropods from caves inhabited by insectivorous bats in red; C. Arthropods from caves without bats in gray.

The Venn diagrams below illustrate the overlap of arthropod taxa among caves inhabited by frugivorous bats, insectivorous bats, and caves without bats, by three taxonomic ranks: order, family, and species (Figure 6). The non-arachnid order dataset showed 16 orders in frugivorous caves, 11 in insectivorous caves, and 14 in caves without bats. Five orders are unique to caves inhabited by frugivorous bats (Embioptera, Polydesmida, Scutigeromorpha, Orthoptera, Psocoptera), only one order is unique to caves inhabited by insectivorous bats (Diplura), and three orders are unique to caves without bat colonies (Heteroptera, Trichoptera and Zygentoma). Eight orders were found in all cave types (Blattodea, Coleoptera, Collembola, Diplopoda, Diptera, Hymenoptera, Isopoda and Lepidoptera) (Figure 6a). The non-arachnid species dataset showed 61 species in frugivorous caves, 45 in insectivorous caves, and 36 in caves without bats; of them, 42 species were unique to caves inhabited by frugivorous bats, 24 species and morphospecies were unique to caves inhabited by insectivorous bats, and 16 species and morphospecies were unique to caves without bat colonies. Only eight species and morphospecies belonging to the following arthropod orders were common to all cave types: Coleoptera, Diptera (family Dolichopodidae), Blattodea (*Polyphaga aegyptiaca*), Collembola, Diplopoda (MO 3), and Isopoda (Porcellionidae) (Figure 6b).

The arachnid family dataset showed fourteen families in frugivorous caves, eleven in insectivorous caves, and ten in caves without bats, with six shared families (Agelenidae, Sicariidae, Argasidae, Theridiidae, Dysderidae, and Trombidiformes); five families were unique to caves inhabited by frugivorous bats (Gnaphosidae, Oribatida, Uloboridae, Uropodina and Chernetidae), two families were unique to caves inhabited by insectivorous bats (Eukoeneniidae and Leptonetidae), and two families were unique to caves without bat colonies (Nesticidae and Trogulidae) (Figure 6c). The arachnid species and morphospecies dataset showed 27 species in frugivorous caves, 17 in insectivorous caves, and 17 in caves without bats, with 16 species unique to caves inhabited by frugivorous bats, 7 species unique to caves inhabited by insectivorous bats, and 10 species unique to caves without bat colonies, with 4 species (*Harpactea* sp., *Loxosceles rufescens*, *Ornithodoros tholozani*, and *Araneae* sp.) common to all caves (Figure 6d).

### 3.3. Taxonomic Composition of Arthropod Assemblages Across Cave Types

**Figure 7:**
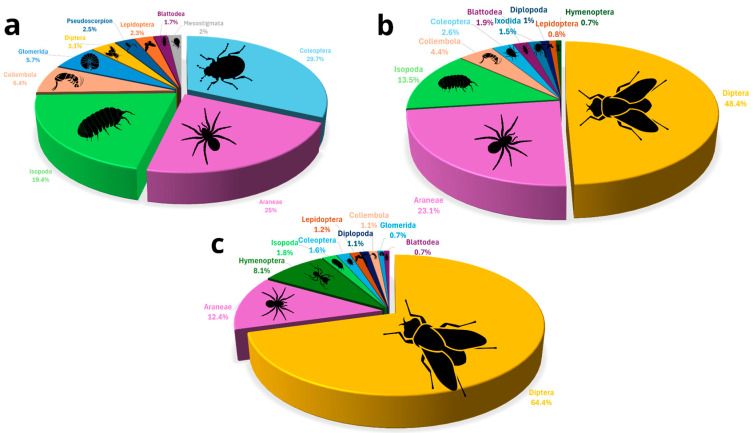
Percentages of the ten most common arthropod orders across caves: (**a**) caves with frugivorous bat colonies, (**b**) caves with insectivorous bat colonies, and (**c**) caves without bats. Colors denote arthropod orders, with labels indicating the ten most common orders in each panel.

In caves inhabited by frugivorous bats, predators accounted for more than 25% of the assemblage, with spiders representing the dominant group. Decomposers (including isopods, millipedes, collembola, and some beetles) made up over 33% of the fauna, whereas flies (Diptera) were comparatively scarce. In insectivorous bat caves, predators also accounted for more than 25% of the assemblage, with spiders representing the dominant group. Decomposers (including isopods, millipedes, collembola, and some beetles) composed roughly 25% of the assemblage, while flies made up more than 40% of the arthropods. Finally, in caves without bats, spiders remained the main predators; however, flies overwhelmingly dominated the community, representing nearly 60% of all arthropods, while decomposers such as isopods and other groups were notably less abundant (Figure 7).

### 3.4. Environmental Drivers of Arachnid Assemblage Composition

**Figure 8:**
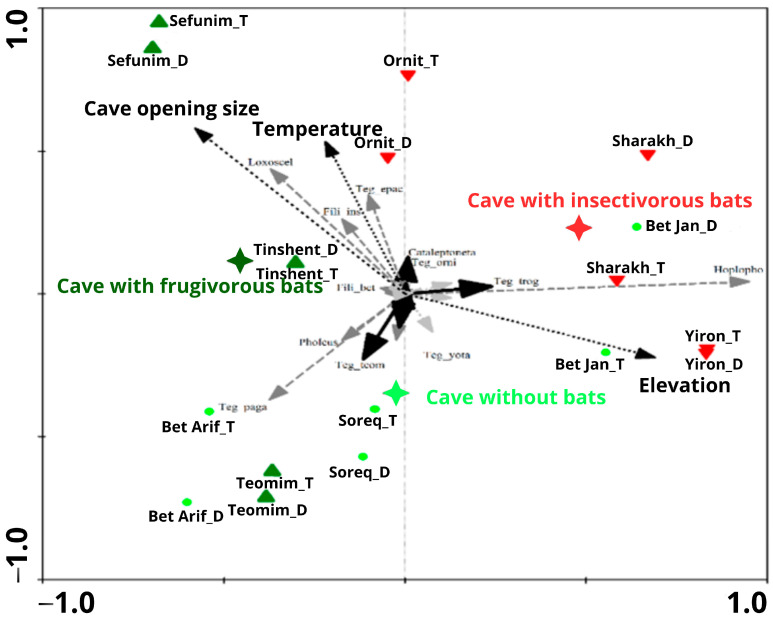
CCA ordination graph of the first and second axes, testing nine caves with three different types of bat colonies (frugivorous, insectivorous, and without bats) from two zones (twilight and dark) and troglobite/troglophile arachnid species. The significant explanatory variables (elevation, temperature, and cave opening size) are plotted on the graph, as are the cave and species names. Cave types are represented as follows: cave with frugivorous bats: dark green triangle; cave with insectivorous bats: red triangle; cave without bats: lime green dots. Troglobite species are indicated by black arrows, troglophile species by gray arrows, and visitor species by light gray arrows. Nominal explanatory variable is shown by a black star, and continuous explanatory variables by arrows with a dashed line.

Arachnid assemblage composition was significantly affected by several environmental variables: elevation (CCA; *p*-value = 0.0008, F-ratio = 2.72), caves with insectivorous bat colonies (*p*-value = 0.0002, F-ratio = 2.85), cave opening size (*p*-value = 0.0088, F-ratio = 2.03), and temperature (*p*-value = 0.0010, F-ratio = 2.37). Caves with frugivorous bat colonies only marginally affected spider assemblage composition (CCA; *p*-value = 0.0752, F-ratio = 1.59). All environmental variables taken together explained 58% of the variance in spider assemblage composition (Figure 8).

## 4. Discussion

Our findings point to bat guano as the primary driver of arthropod assemblage composition in Levantine caves, shaping total abundance as well as species richness across ecological zones and microhabitats. Cave type (with or without bat colonies) emerged as the dominant structuring factor: caves hosting bat colonies exhibited higher total arthropod abundance and species richness than bat-free caves. Arthropod abundance and species richness was higher in frugivorous bat caves than in insectivorous bat caves. Within this hierarchy, ecological zonation had a further modulating effect on assemblages, with arachnid troglobite richness being highest in the dark zone, particularly in insectivorous bat caves. Overall, these patterns are well aligned with our stated initial hypotheses, particularly the expectation that differences in guano type and deposition zone would drive distinct assemblage structures. In addition, the strong microhabitat and zone effects are consistent with our predictions regarding spatial microhabitat within cave environments.

Our results support those of previous studies showing that organic inputs such as bat guano are key drivers of functional biodiversity in nutrient-limited hypogean environments, providing nutrient sources and supporting structurally complex decomposer and predator communities [2,39]. In caves where guano accumulates, these nutrient-rich deposits create ecological hotspots that contrast with the surrounding oligotrophic subterranean matrix. Our findings also add to prior evidence that guano patches reorganize trophic roles [7,28,30], demonstrating that guano type and colony presence systematically modulate the proportional dominance of predators, decomposers, and flies at the cave scale. Frugivorous bat systems support more diverse detrital communities and richer predator assemblages [20], while insectivorous bat and bat-free caves support more Diptera-dominated assemblages. The interaction between cave type (with or without bat colonies) and ecological zone was significant, affecting total abundance, species richness, assemblage composition, and troglobitic richness. Together, these findings highlight the fact that guano-driven subsidy influences the structure of local communities, reinforcing the role of bats in hypogean environments.

At finer spatial scales, microhabitat heterogeneity acted as an additional filter, with certain taxa exhibiting clear microhabitat preferences. Microhabitat selection has been shown to play a crucial role in reducing interspecific competition, allowing for multiple spider species to coexist through spatial niche partitioning [40]. Generally speaking, the presence of guano per se and its spatial distribution within caves, whether deposited near entrances, in twilight zones, or deep in dark zones, significantly influenced arthropod community structure. Guano deposited deeper in dark zones was associated with a higher proportion of troglobitic species and a distinct assemblage of arachnids, as opposed to guano near entrances; this highlights the combined role of cave morphology, bat behavior, and guano deposition patterns in shaping these communities [19,41].

## 5. Conclusions

Overall, our results highlight the central role of guano, its source, and its spatial distribution within caves in shaping arthropod communities in Levantine caves. Evaluating cave type and deposition zones within the same modeling framework, captures their interactive effects, linking resource quality, spatial deposition, zonation, and microhabitat filtering as defining factors in hypogean habitats. Practically, conservation and monitoring should consider bat species composition and guano deposition patterns across zones, given their cascading implications for detrital processing, predator persistence, and the long-term maintenance of dark-adapted specialists. Several limitations should be considered, however, as our sampling represents a single temporal window and may not have captured seasonal variation in guano deposition or arthropod activity. Additionally, unmeasured microbial processes and fine-scale cave environmental heterogeneity may contribute to unexplained variation. Moreover, the presence of some arthropod orders in only a single cave type (inhabited by frugivorous bat colonies, insectivorous bat colonies, or without bat colonies) is still not understood. Future work incorporating temporal and microbial components would help refine our understanding of the data. In the current publication, we focus on bat guano; however, more research should be conducted on the importance of other allochthonous organic subsidies from other sources than of bats.

## Figures and Tables

**Table 1:** Total abundance and percentage of all arthropods from the most abundant orders (those with more than 100 individuals), which account for 90% of the total sample (both collected and released—see text).

Order	Total Abundance and % of All Arthropods in Caves Housing Different Bat Colony Types			Total Abundance and % of All Arthropods in All Cave Types
	Frugivorous Bats	Insectivorous Bats	Caves Without Bats	
Diptera	51 (3.2%)	697 (48.4%)	951 (69.4%)	1699 (38.5%)
Araneae	327 (20.4%)	333 (23.1%)	170 (12.4%)	830 (18.8%)
Coleoptera	476 (29.7%)	38 (2.6%)	22 (1.6%)	536 (12.2%)
Isopoda	312 (19.5%)	194 (13.5%)	25 (1.8%)	531 (12.0%)
Collembola	103 (6.4%)	64 (4.4%)	15 (1.1%)	182 (4.1%)
Hymenoptera	19 (1.2%)	10 (0.7%)	111 (8.1%)	140 (3.2%)
Glomerida	92 (5.7%)	0 (0%)	10 (0.7%)	102 (2.3%)

**Table 2:** Statistical analysis of environmental factors affecting cave arthropod and arachnid diversity. Significant *p*-values are in bold.

		Arthropod Species Abundance		Arthropod Species Richness		Arachnid Troglophile Species Richness		Arachnid Troglobite Species Richness	
Source	DF	Chi-Square	*p*	Chi-Square	*p*	Chi-Square	*p*	Chi-Square	*p*
Ecological zone	1	871.43811	**0.0001**	18.460635	**0.0001**	10.635347	**0.0011**	1.0432444	**0.0443**
Cave type	2	134.9522	**0.0001**	53.863126	**0.0001**	15.567073	**0.0004**	10.366709	**0.0056**
Zone × Cave type	2	211.6879	**0.0001**	2.8472486	0.2408	4.7598304	0.0926	8.1413147	**0.0171**
Microhabitats	2	79.499781	**0.0001**	30.660839	**0.0001**	3.8671121	0.1446	1.5213041	0.4674

## Data Availability

The original contributions presented in this study are included in the article/Supplementary Material. Further inquiries can be directed to the corresponding authors.

## Supplementary Materials

The following supporting information can be downloaded at https://www.mdpi.com/article/10.3390/insects17010118/s1, Table S1: Raw arthropod cave database.

## Abbreviations

The following abbreviations are used in this manuscript:

ANOVA	Analysis of variance
CCA	Canonical Correspondence Analysis
df	Degrees of freedom
GLMM	Generalized Linear Mixed Model
GIS	Geographic Information System
sp.	Undetermined species within a genus

## Appendix A

**Table A1:** List of the nine caves sampled in this study and their environmental variables.

Cave Type	Cave Name	N	E	Elevation	Cave Length Estimate	Cave Entrance Size	Zone	Temperature	Humidity
Caves with frugivorous bat colonies	(1) Sefunim	32.7370	34.9784	126	50	104	Twilight	26.3	0.84
							Dark	25.7	0.823
	(2) Tinshemet	31.9994	34.9681	100	43	97	Twilight	29	0.76
							Dark	29	0.79
	(3) Te’omim	31.7262	35.0217	375	134	128	Twilight	17.3	0.795
							Dark	16.7	0.86
Caves with insectivorous bat colonies	(4) Yir’on	33.0679	35.4665	528	150	79	Twilight	21	0.961
							Dark	20.9	0.875
	(5) Sharakh	33.0740	35.2378	299	85	99	Twilight	25.1	0.81
							Dark	28	0.223
	(6) Ornit	32.7566	34.9897	192	66	71	Twilight	23	0.83
							Dark	21.1	0.894
Caves without bat colonies	(7) Bet Jan	32.9615	35.3951	927	33.5	105	Twilight	24.3	0.824
							Dark	22.2	0.88
	(8) Bet A’rif	32.0026	34.9642	95	45	73	Twilight	16.2	0.823
							Dark	19.1	0.74
	(9) Soreq	31.7560	35.0227	405	80	10	Twilight	21.7	0.984
							Dark	20.6	0.919

## References

[1] Barr T.C., Holsinger J.R. (1985). Speciation in cave faunas. Annu. Rev. Ecol. Syst..

[2] Gibert J., Deharveng L. (2002). Subterranean ecosystems: A truncated functional biodiversity. BioScience.

[3] Mammola S. (2019). Finding answers in the dark: Caves as models in ecology fifty years after Poulson and White. Ecography.

[4] Gavish-Regev E., Aharon S., Armiach Steinpress I., Seifan M., Lubin Y. (2021). A primer on spider assemblages in Levantine caves: The neglected subterranean habitats of the Levant—A biodiversity mine. Diversity.

[5] Cuff J.P., Aharon S., Armiach Steinpress I., Seifan M., Lubin Y., Gavish-Regev E. (2021). It’s all about the zone: Spider assemblages in different ecological zones of Levantine caves. Diversity.

[6] Manenti R., Lunghi E., Ficetola G.F. (2015). The distribution of cave twilight-zone spiders depends on microclimatic features and trophic supply. Invertebr. Biol..

[7] Howarth F.G., Moldovan O.T., Moldovan O.T., Kováč Ľ., Halse S. (2018). Where cave animals live. Cave Ecology.

[8] Tobin B., Hutchins B., Schwartz B. (2013). Spatial and temporal changes in invertebrate assemblage structure from the entrance to deep-cave zone of a temperate marble cave. Int. J. Speleol..

[9] Poulson T.L., White W.B. (1969). The cave environment: Limestone caves provide unique natural laboratories for studying biological and geological processes. Science.

[10] Howarth F.G. (1993). High-stress subterranean habitats and evolutionary change in cave-inhabiting arthropods. Am. Nat..

[11] Sket B. (2008). Can we agree on an ecological classification of subterranean animals?. J. Nat. Hist..

[12] Culver D.C., Pipan T. (2019). Adaptations to subterranean life. The Biology of Caves and Other Subterranean Habitats.

[13] Resende L.P.A., Bichuette M.E. (2016). Sharing the space: Coexistence among terrestrial predators in Neotropical caves. J. Nat. Hist..

[14] Kováč Ľ., Moldovan O.T., Kováč Ľ., Halse S. (2018). Caves as oligotrophic ecosystems. Cave Ecology.

[15] Engel A.S. (2019). Chemolithoautotrophy. Encyclopedia of Caves.

[16] Wischer D., Kumaresan D., Johnston A., El Khawand M., Stephenson J., Hillebrand-Voiculescu A.M., Chen Y., Murrell J.C. (2015). Bacterial metabolism of methylated amines and identification of novel methylotrophs in Movile Cave. ISME J..

[17] Cardoso P. (2012). Diversity and community assembly patterns of epigean vs. troglobiont spiders in the Iberian Peninsula. Int. J. Speleol..

[18] Chen B., Wise D.H. (1999). Bottom-up limitation of predaceous arthropods in a detritus-based terrestrial food web. Ecology.

[19] Simon K.S., Benfield E.F., Macko S.A. (2003). Food web structure and the role of epilithic biofilms in cave streams. Ecology.

[20] Ferreira R.L., Martins R.P. (1999). Trophic structure and natural history of bat guano invertebrate communities, with special reference to Brazilian caves. Trop. Zool..

[21] Emerson J.K., Roark A.M. (2007). Composition of guano produced by frugivorous, sanguivorous, and insectivorous bats. Acta Chiropterologica.

[22] Abd Rahman S.-S.N.F., Tawie Tingga R.C., Mohamad Bukhori M.F., Abdullah S.M.A.A. (2023). A brief review of the nutritive value and chemical components of bat guano and its potential use as a natural fertiliser in agriculture. Borneo J. Resour. Sci. Technol..

[23] Gnaspini P., White W.B., Culver D.C. (2012). Guano communities. Encyclopedia of Caves.

[24] Mammola S., Cardoso P., Ribera C., Pavlek M., Isaia M. (2018). A synthesis on cave-dwelling spiders in Europe. J. Zool. Syst. Evol. Res..

[25] Trajano E., Bichuette M.E. (2010). Diversity of Brazilian subterranean invertebrates, with a list of troglomorphic taxa. Subterr. Biol..

[26] Dippenaar-Schoeman A.S., Myburgh J.G. (2009). A review of the cave spiders (Arachnida: Araneae) from South Africa. Trans. R. Soc. S. Afr..

[27] Turbanov I.S., Palatov D.M., Golovatch S.I. (2016). The state of the art of biospeleology in Russia and other countries of the former Soviet Union: A review of the cave (endogean) invertebrate fauna. 2. Arachnida—Acknowledgments. Entomol. Rev..

[28] Ferreira R.L., Prous X., Martins R.P. (2007). Structure of bat guano communities in a dry Brazilian cave. Trop. Zool..

[29] Webster J.M., Whitaker J.O. (2005). Study of guano communities of big brown bat colonies in Indiana and neighboring Illinois counties. Northeast Nat..

[30] Ferreira R.L., Martins R.P. (1998). Diversity and distribution of spiders associated with bat guano piles in Morrinho Cave (Bahia State, Brazil). Divers. Distrib..

[31] Dainelli L., Martínez A., Serena F., Gammuto L., Graco-Roza C., Langeneck J., Mammola S., Petroni G. (2025). Macroinvertebrate diversity patterns in a guano-rich temperate cave. Biodivers. Conserv..

[32] Smith T.M., Smith R.L., Wilbur B. (2012). Decomposition and nutrient cycling. Elements of Ecology.

[33] Gloor D., Blick T., Nentwig W., Kropf C., Hänggi A. (2010). Spiders of Europe. Natural History Museum Bern. https://www.araneae.nmbe.ch.

[34] Roberts M.J. (2016). Spiders of Britain and Northern Europe.

[35] Warburg S., Aharon S., Armiach Steinpress I., Sharma P.P., Harms D., Gavish-Regev E. (2023). Pseudoscorpions of Israel: Annotated checklist and key, with new records of two families (Arachnida: Pseudoscorpiones). Taxonomy.

[36] ter Braak C.J.F. (1986). Canonical correspondence analysis: A new eigenvector technique for multivariate direct gradient analysis. Ecology.

[37] ter Braak C., Šmilauer P. (2020). Canoco—Software for Ordination.

[38] ter Braak C., Šmilauer P. (2018). Canoco Reference Manual and User’s Guide: Software for Ordination.

[39] Magalhaes I.L.F., Aharon S., Ganem Z., Gavish-Regev E. (2022). A new semi-cryptic *Filistata* from caves in the Levant with comments on the limits of *Filistata insidiatrix* (Forsskål, 1775) (Arachnida: Araneae: Filistatidae). Eur. J. Taxon..

[40] Aharon S., Ballesteros J.A., Gainett G., Hawlena D., Sharma P.P., Gavish-Regev E. (2023). In the land of the blind: Exceptional subterranean speciation of cryptic troglobitic spiders of the genus *Tegenaria* (Araneae: Agelenidae) in Israel. Mol. Phylogenetics Evol..

[41] da Rocha Melo L.M., Ferreira R.L., Silva M.S. (2025). A review of the factors influencing invertebrate community structure in subterranean habitats. Community Ecol..

